# Effect of ketofol versus propofol as an induction agent on ease of laryngeal mask airway insertion conditions and hemodynamic stability in pediatrics: an observational prospective cohort study

**DOI:** 10.1186/s12871-019-0711-0

**Published:** 2019-03-20

**Authors:** Bacha Aberra, Adugna Aregawi, Girmay Teklay, Hagos Tasew

**Affiliations:** 1 Aksum University, PO box 298, Aksum City, Tigray Ethiopia; 2 Addis Ababa University, PO box 811/1000, Addis Ababa, Ethiopia

**Keywords:** Hemodynamics, Ketofol, Laryngeal mask airway insertion, Propofol

## Abstract

**Background:**

Laryngeal mask airway is a supraglottic airway device which has led to a fundamental change in the management of modern general anesthesia. In the present study; we evaluated the laryngeal mask airway insertion conditions and hemodynamic changes comparing ketamine-propofol mixture (ketofol) with propofol. The study was to compare the ketamine–propofol mixture (ketofol) with propofolon the ease of laryngeal mask airway insertion conditions and hemodynamic effects for induction of general anesthesia.

**Methods:**

One hundred twenty pediatric patients were recruited and assigned to two groups (60 each). Group KP = ketofol, group P = propofol. Insertion conditions were compared using a Chi-square test while hemodynamic variables were compared using the independentt-test. Statistical significance was stated at *p*-value< 0.05.

**Results:**

Laryngeal mask airway insertion summed score was nearly similar between the two groups. Mean blood pressure and heart rate were maintained higher in ketofol group while a significant drop was observed in the propofol group. The time from the Laryngeal mask airway placement to the return of spontaneous ventilation was significantly longer in propofol group (240 s [range = 60–360 s]) compared with ketofol group (180 s [range = 30–320 s]) (*p* = 0.005).

**Conclusions:**

Laryngeal mask airway insertion condition summed score was comparable in both ketofol and propofol group. Ketofol provided equivalent laryngeal mask airway insertion conditions while maximizing hemodynamics and minimizing apnea time. Ketofol can be used as an alternative to propofol for laryngeal mask airway insertion in pediatrics.

## Background

The most important duty of an anesthetist is the management of airway to deliver sufficient ventilation to the patient by securing airway while general anesthesia is administered. As such, no anesthesia is safe unless meticulous efforts are devoted to maintain an intact and functional airway [1, 2]. Effective insertion of the LMA entails optimum anesthetic depth to elude undesirable airway reflexes such as swallowing, gagging, coughing or involuntary movements to severe problems such as laryngospasm [3, 4].

Adequate anesthetic induction situations are paramount delivered by propofol compared to other intravenous induction agents [4]. Nevertheless, when propofol is used as a single induction agent without premedication, doses greater than 3 mg/kg is necessary for smooth LMA insertion [5, 6]. On the other hand, increased propofol doses are not required as undesirable cardio-respiratory depression is dose-dependent [7, 8]. Several combinations of pharmacological agents have been introduced to decrease the hemodynamic instability in anesthesia [9, 10]. Ketamine is well known for its airway reflexes maintaining activity and sympathetic cardiorespiratory stimulant so as to causes little or no cardiorespiratory depression and unlike propofol has pain relieving properties [11, 12].

Hemodynamic stability can be maintained using a combination of ketamine and propofol (ketofol), as there is additive effect of Gamma-aminobutyric acid (GABA) agonism by propofol and N-Methyl-D-Aspartate (NMDA) antagonism by ketamine leading to lesser doses of propofol required along with ketamine [13]. The effectiveness of the two agents, propofol and ketamine, may provide the best induction agent with favorable hemodynamics and decreased side effects attributed to either drug as clinical effects of propofol and ketamine seem to be complementary [14].

Therefore the finding of this research will help anesthesia professionals to provide safe and effective alternative induction agent for better LMA insertion conditions and improved hemodynamic stability. It also helps health administrators to work on quality improvement, enhancing good patient outcome, supplying cost-effective anesthetic drugs with the better patient outcome and enhancing income generation and cost reduction.

## Methods

### Study objective

The aim of this study was to compare the effect of the ketamine-propofol mixture (ketofol) and propofol on the insertion conditions of laryngeal mask airway and hemodynamic stability in pediatrics.

### Study design

An observational prospective cohort study was employed from Jan 25-March, 25, 2017 after ethical approval (No-11/2009, Dec 1, 2016) was obtained from the Addis Ababa University Ethical committee.

### Study setting

This study was conducted at Menelik-II Hospital. Menelik II hospital is one of the largest hospitals in the country. Menelik-II Hospital is now the main health provider center that offers high-quality comprehensive health services to the patient from all over the region of Ethiopia and there are two main operation departments, from which ophthalmic operation room has six operation tables. One of these is pediatric operation room table, which on average, 1920 pediatric patients operated under general anesthesia per year. The study was conducted from January 25–March 25, 2017.

### Study participants

Patients of ASA class I and II, age ranging from 2 to 15 years and undergoing elective surgical procedures under GA using LMA were included in the study. Patients with hyper-reactive airway disease anticipated difficult airway, on regular sedatives and on β-blockers were excluded from the study.

### Study variables

In this study, the dependent variables were ease of LMA insertion and hemodynamic changes. The independent variables were socio-demographic and operative data (Age, Sex Weight, ASA, and Mallampati class) and another exposure variable (a type of anesthesia drugs used (ketofol vs. propofol)) Table 1.

**Table 1:** operational definitions

Operational Definitions		
Apnea	The absence of spontaneous respiration for < 20 s after induction	
Ease of insertion	Easy	No adverse response, i.e., gagging or coughing, movement or laryngospasm
	Difficult	Moderate to severe adverse responses requiring additional boluses of drugs or more than two attempts are required for LMA insertion
Laryngospasm	Complete	when there are laryngeal spasm and no air entry on ventilation
	Incomplete	when there is laryngeal spasm but there is air entry
Coughing	Slight	coughing which can occur immediately after LMA and subside by itself
	Gross	coughing which needs deepening of anesthesia to be relieved
Gagging [23]	Slight	Gagging which stays for short seconds can relieve on its own
	Gross	Gagging which needs deepening of anesthesia to be relieved
Patient movement	Slight	Movement from small muscles which can allow insertion of LMA without an additional dose of the drugs
	Gross	The movement which cannot be relieved without an additional dose of the drugs
Insertion condition summed score	Summing the insertion score for each patient then totaling the score for all patients in the groups and taking the mean	

### Sample size and sampling techniques determination

StatCalc EPI info 7.1.1 (Fleiss) was used based on ease of LMA insertion conditions among two groups to calculate the sample size for each group. The following assumptions were considered to estimate the sample size required for the study. A 95% confidence level and 80% power, equal sample size for two groups, a proportion of subjects with poor insertion conditions were 41.66 and 18.33% in propofol (unexposed) and ketofol (exposed) group respectively in a recent study [15, 16]. A total of 60 ASA I and II pediatric patients age 2–15 were assigned to each group.

## Sampling technique

From situational analysis mean of midyear population was used to get a total number of ophthalmic pediatric patients who underwent operation under general anesthesia using LMA in 2 months duration. The midyear population from the situational analysis was 960. So, the size of the population in 2 months was 960 divided by three gives us 320. The study participants were selected using a systematic sampling technique every two participants from daily operation schedule list until the required sample size was obtained. The first study participants were selected by lottery method. We spent two extra weeks to reach the number of propofol group is equal to ketofol group to get an equal sample size in both groups.

### Intraoperative procedure

After preoperative preparation, patients were shifted to the operation room, standard monitoring applied as routine. Baseline vitals were recorded and I.V. fluids were administered. Patients were preoxygenated with 6 L/min of Oxygen via a face mask, for 3 min and given injection atropine 0.02 mg/kg I.V. and fentanyl 1 μg/kg I. V prior to induction as the standard of care.

LMA insertion was performed 60 s after induction of anesthesia [1]. Following insertion, the position of LMA was assessed by observing movement of chest and reservoir bag through use of both spontaneous and assisted ventilation. After successful insertion of LMA, patients were allowed to breathe spontaneously. Assisted manual ventilation provided when the apnoea period is longer than 20 s from the time of LMA insertion to ensure that SpO2 remained above 95%. Manual ventilation was stopped when sufficient spontaneous respiration returned. Thereafter, anesthesia was maintained with isoflurane 2% and oxygen 100% with a flow rate of 3 L/min.

The patients were either induced with ketofol (0 .5mg/kg of ketamine plus 3 .0mg/kg of propofol) or 3 .5mg/kg or propofol alone. If the patients respond to stimulus after induction, further increments of propofol 0.5–1 mg/kg were given until loss of consciousness and loss of eyelash reflex in either technique. All patients who were exposed to either ketofol or propofol were compared to see different outcomes of both agents as an induction agent on ease of laryngeal mask airway insertion and hemodynamic stability. Insertion condition was graded by the same anesthetist who performs the procedure as [9].

- Mouth opening: 1 – Full, 2 – Partial, 3 – Nil
- Coughing: 1 – Nil, 2 – slight, 3 – gross
- Swallowing: 1 – Nil, 2 – slight, 3 – gross
- Movement: 1 – Nil, 2 – slight, 3 – gross
- Laryngospasm: 1 – Nil, 2 – Mild, 3– Severe
- Ease of LMA insertion: 1-Easy, 2-Difficult, 3- Impossible

Mean blood pressure and heart rate were recorded one minute before induction (baseline), immediately after induction, immediately after LMA insertion, then at every minute for up to 3 min. The duration of apnoea was recorded via a digital timer as the time from the end of induction of anesthesia until the return of adequate spontaneous ventilation. Afterward, all patients who were scheduled for ophthalmic surgical operation under general anesthesia with LMA were enrolled in the study and assigned to either ketofol or propofol group randomly. Our study used those patients induced with propofol as a cohort group, where the same checklist was used to observe the case.

### Data collection technique and instrument

Data were collected using a pretested observational checklist. Data collectors were three bachelor degree holder anesthetist and they supervised by one master degree holder anesthetist. All anesthetists participating in the study including anesthetists who inserts the LMAs and administers the medications had at least 2 years of experience in conducting anesthesia.

### Data quality assurance

Before recruiting patients into the study, training and orientation about the objective and process of data collection were provided by the principal investigator. To ensure the quality of data, a pre-test of the checklist was performed in Cure International Hospital before the actual data collection time. The completed checklist was submitted and reviewed on daily basis. Close supervision and daily information exchange were used as a means to correct problems during the course of data collection.

Consent for the survey was obtained from Addis Ababa University College of health sciences and confidentiality assured to improve the quality of data. Variables were checked by the expert before the actual data collection period for the purpose of consistency.

### Statistical methods

All data were analyzed by SPSS statistical package program (Version 20). Within the groups, the normality of variables was measured using the Shapiro-Wilk test. Differences of numerical data between groups were evaluated using student’s t-test and Mann–Whitney U-test when appropriate. Categorical data were analyzed with the Chi-Square test. A *p* value of < 0.05 with the power of 80% was regarded as statistically significant.

## Results

### Socio-demographic features and operative conditions

A total of 120 patients were enrolled and none were excluded from the study as there was no incidence of failed LMA insertion. There were no statistically significant differences in age, sex, weight, and ASA or mallampati class between groups [Table 2].

**Table 2:** Socio-demographic features of the patients

Patient characteristics	Group KP (*n* = 60)	Group P (*n* = 60)	*P*-value
Age in years (median, IQR^a^)	5.5 (3–9)	7 (4–11)	0.18
Gender (n, %)			1.00
Male	34 (56.7)	35 (58.3)	
Female	26 (43.3)	25 (41.7)	
Weight (median, IQR^*^) (in Kgs^b^)	19.5 (14–25)	26 (15–30)	0.14
ASA (n, %)			0.611
I	57 (95.0)	59 (98.3)	
II	3 (5.0)	1 (1.7)	
Mallampati class (n, %)			0.756
Can’t be assessed^c^	18 (30)	16 (26.7)	
I	40 (66.7)	43 (71.7)	
II	2 (3.3)	1 (1.7)	

^a^ = Interquartile range, ^b^ = kilograms, ^c^ = (< 4 years of age, uncooperative)

## Comparison of ease of insertions conditions

In 12 (20%) of the propofol group patients, additional 0.5–1 mg/kg) propofol was required as compared to nine (15%) of those in the ketofol group. However, no significant difference was noted between the two groups (*p* = 0.631). The time from the LMA placement to the return of spontaneous ventilation was significantly longer in propofol group (240 s [range = 60–390 s]) compared with a ketofol group (180 s [range = 30–380 s]) (*p* = 0.005), expressed in median and range respectively. The LMA was inserted successfully and positioned correctly on the first attempt in 95% of patients receiving ketofol compared with 96.67% in patients receiving propofol [Table 3].

**Table 3:** Requirement of Additional propofol, duration of apnea and attempts of LMA

	Group KP	Group P	*p*-value
The requiredtop-up dose of propofol (n, %)	9 (15)	12 (20)	0.631
Duration of apnea (seconds)	180^a^(30–380^b^)	240^a^(60–390^b^)	0.005
Attempts of LMA insertion(1/2/3)	57/3/0	58/2/0	0.648

^a^=Median, ^b^ = range

LMA insertion summed score was nearly similar between the two group, which were statistically insignificant (*p* = 0.511). No patient developed laryngospasm in the ketofol group while 2 patients (3.33%) developed partial laryngospasm in propofol group but not statistically significant (*p* = 0.154) [Table 4].

**Table 4:** Comparison of insertion conditions of LMA between the ketofol and propofol groups

Assessment grades	Group KP	Group P	*p* value
Mouth opening (Full / Partial/None)	54/6/0	53/7/0	0.769
Coughing or gagging (Nil / Slight/Gross)	54/6/0	52/7/1	0.573
Swallowing (Nil / Slight/Gross)	55/5/0	54/5/1	0.604
Laryngospasm (Nil / Partial/Complete)	60/0/0	58/2/0	0.496
Ease of LMA insertion (easy/difficult/impossible)	59/1/0	58/2/0	0.956
Head or limbs movement (Nil / Slight/Gross)	57/3/0	58/2/0	0.648
Insertion condition summed score	6.35 (6–10)	6.48 (6–13)	0.607

## Comparison of hemodynamic characteristics

**Fig. 1:**
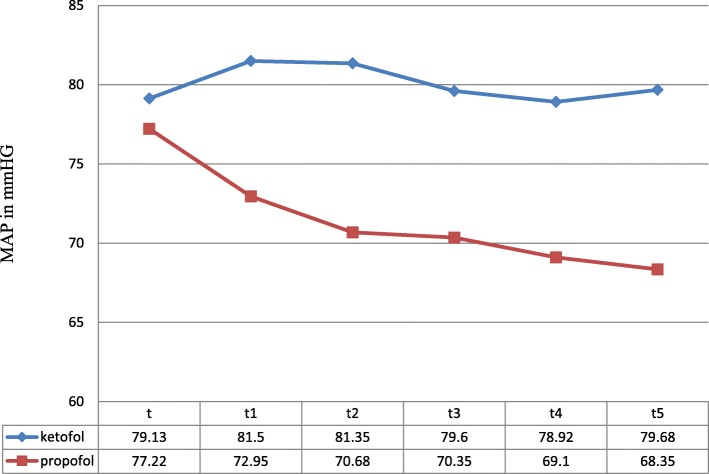
Changes in mean arterial pressure between ketofol and propofol group. NB: t, baseline, t1, immediately following induction of anesthesia, t2, immediately after LMA placement, t3, t4, and t5,1,2 and 3 min after LMA placement

Patients in both groups were comparable with respect to preoperative baseline hemodynamic conditions. The mean arterial pressure difference between the group is not significantly different before induction (*p*-value = 0.263; is insignificant). With the induction of anesthesia, a significant drop in mean arterial blood pressure was observed in propofol group from baseline while in the ketofol group, there was a rise in mean arterial pressure at all measurement times (*P* < 0.001) [Fig. 1]. Maximum mean blood pressure was 81.5 ± 11.02 mmHg with a ketofol group seen immediately after induction [Table 5].

**Table 5:** Comparing the Mean of data on mean arterial blood pressure between ketofol and propofol groups

	Mean arterial pressure (in mmHg)		*P* value
	Group KP (Mean ± SD)	Group P (Mean ± SD)	
Baseline MAP	79.13 ± 8.96	77.22 ± 9.69	0.263
Immediately after induction	81.50 ± 11.02	72.95 ± 12.349	< 0.001
Immediately after LMA insertion	81.35 ± 11.339	70.68 ± 11.620	< 0.001
One minute after LMA insertion	79.60 ± 11.036	70.35 ± 10.844	< 0.001
Two minute after LMA insertion	78.92 ± 11.794	69.10 ± 10.188	< 0.001
Three minute after LMA insertion	79.68 ± 11.978	68.35 ± 9.295	< 0.001

**Fig. 2:**
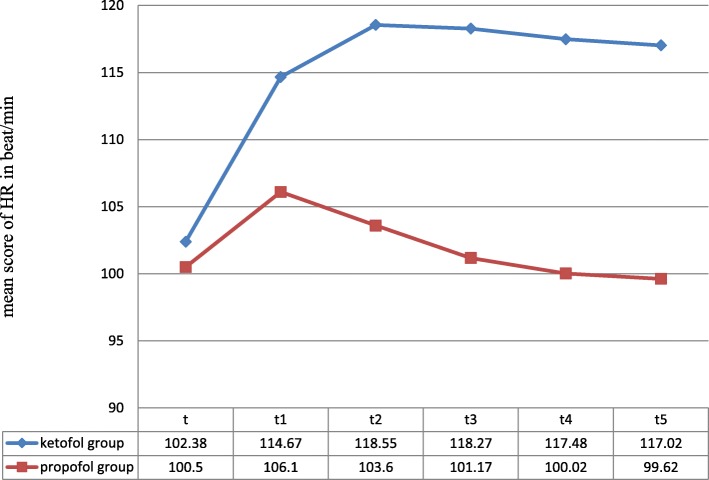
Changes in heart rate between ketofol and propofol group. NB: t, baseline, t1, immediately following induction of anesthesia, t2, immediately after LMA placement, t3, t4, and t5,1,2 and 3 min after LMA placement

Preoperatively there was no statistically significant difference (*p*-value> 0.05) between the heart rate of both groups. *P* values at all levels after induction were < 0.05 and statistically significant. With the induction of anesthesia, a significant rise in heart rate was observed in the ketofol group from baseline while in propofol group, there was a drop in heart rate at all measurement times (*P* < 0.05) [Fig. 2]. The increment in heart rate was seen immediately after induction compared with baseline. Maximum heart rate was 118.55 ± 24.86 beat/min with a ketofol group seen immediately after LMA insertion. Minimum heart rate (99.62 ± 25.071) was seen in propofol group 3 min after LMA insertion [Table 6].

**Table 6:** Comparing the Mean of data on heart rate between ketofol and propofol group

	Heart rate (beat per minute)		*P* value
	Group KP (Mean ± SD)	Group P (mean ± SD)	
Baseline heart rate	102.38 ± 16.368	100.50 ± 17.070	0.539
Immediately after induction	114.67 ± 21.972	106.10 ± 21.802	0.034
Immediately after LMA insertion	118.55 ± 24.863	103.60 ± 23.449	< 0.001
One minute after LMA insertion	118.27 ± 22.823	101.17 ± 24.668	< 0.001
Two minute after LMA insertion	117.48 ± 20.994	100.02 ± 25.780	< 0.001
Three minute after LMA insertion	117.02 ± 21.246	99.62 ± 25.071	< 0.001

## Discussion

In our study, LMA insertion summed score for ketofol and propofol group were nearly similar. This result coincides with the studies done by Goh et al. in their study of randomized double-blind comparison of ketamine-propofol, fentanyl-propofol and propofol-saline on hemodynamics and laryngeal mask airway insertion conditions [17].

In our study, in ketofol group, we observed a decrease in the requirement of an additional dosage of propofol for induction, although it was not statistically significant (*p* > 0.05). Consistent with our results, many researchers observed that there was a significant decrease in additional requirement of propofol for induction, loss of consciousness and LMA insertion in ketofol group than propofol group. This less requirement of additional propofol dose is due to the combined effect of ketamine and propofol at both hypnotic and anesthetic endpoints [2, 12, 18]. However, the reason for the insignificant result in our study might have been due to the use of 3.5 mg/kg (high dose) of propofol for induction unlike Yousef et al. [12]. They administered initial 2 mg/kg propofol and incremental doses of propofol until the target level of the Bispectral index of 40 was obtained.

This study also spectacles that, apnea time was significantly longer in propofol group (median = 240 s [range = 60–390 s]) compared with ketofol group (median = 180 s [range = 30–380 s]) (*p* = 0.005). Consistent with our study [19] in their study of comparison of propofol and ketofol on laryngeal tube-suction II circumstances and hemodynamics showed that apnea duration was longer in group P (median = 385 s [range = 195–840 s]) compared with group KP (median = 325.5 s [range = 60–840 s]) but was not statistically significant [19]. In their study, the overall apnea time was higher than ours. This difference might have been due to the use of remifentanil (1 μg/kg) 60 s after pre-oxygenation because remifentanil is known to have prolonged apnea time than fentanyl [13].

A comparative study done in Malaysia to compare the effects of ketamine and midazolam as co-induction agents with propofol for proseal™ laryngeal mask airway insertion showed that the ketamine-propofol combination had a shorter duration of apnoea, better mouth opening, and hemodynamic profile as compared to the combination midazolam-propofol [1].

Another Randomized double-blind comparative study of ketamine-propofol and fentanyl-propofol for LMA insertion in children showed that the conditions of LMA insertion were superior in the combination of ketamine (0.5 mg/kg and propofol than propofol and fentanyl [20].

Hemodynamic parameters can increase 20% after LMA insertion, with an additional 30% after orotracheal intubation [1]. In our study, we observed that ketofol preserved mean arterial pressure at all measurement times while a significant drop in mean arterial blood pressure was seen in the propofol group. Similarly, several studies concluded that ketofol is superior to propofol and propofol–thiopentone mixture because of its better hemodynamic stability [11, 17, 21, 22]. With the induction of anesthesia, a significant rise in heart rate was observed in ketofol group from baseline while in propofol group, there was a drop in heart rate at all measurement times (*P* < 0.05). The cardiovascular stimulant effect of ketofol is desirable especially in pediatric anesthesia while the unduly depressant effect of propofol is unwanted [11, 12].

### Limitations

This study was unable to measure anesthetic depth. Therefore the LMA insertion conditions may have been adversely affected and hemodynamic parameters change might be observed. Use of fentanyl in both groups before induction may have affected the hemodynamic effects of the agents.

## Conclusions

The result of this study showed that LMA insertion condition summed score was comparable in ketofol group and propofol group. There was a decrease in propofol requirement for induction in the ketofol group. There was a more stable MAP picture in the ketofol group when compared to that of propofol group.

## Abbreviations

ASA: American Society of Anesthesiologists
GABA: Gamma-aminobutyric acid
HR: Heart rate
KP: Ketofol
LMA: Laryngeal Mask Airway
MAP: Mean Arterial Pressure
NMDA: N-Methyl-D-Aspartate
P: Propofol
